# Institutional Notes and News

**Published:** 1909-11-06

**Authors:** 


### Institutional Notes and News.

### THE CUMBERLAND INFIRMARY, CARLISLE.

The Governors of the Cumberland Infirmary, Carlisle, have issued an appeal for £5,000 to enable them to finish the alterations sanctioned two years ago. Very sensibly, administrative improvements were first taken in hand, and the new kitchen block and laundry have already been completed. Before the end of the year the Nurses' Home will be occupied. The main feature of the building scheme, however, is the new west pavilion, consisting of a male surgical ward with twelve beds, and above it a children's ward with twenty-four cots. It was decided last month to start on this building. Tenders for the whole scheme, which also included a new casualty operating room, ophthalmic and *x*-ray departments, amounted to £26,000, exclusive of furnishing and architect's fees. These, it is now found, amount to £5,000. By energy and hard work the larger amount has been raised. It is to be hoped the remainder will be, while the contractor's plant is still on the premises.

### NEW HOSPITAL FOR WIMBLEDON.

Mr. D. D. Kirkcaldy, Hon. Secretary, has issued the specifications in the competition for the new general hospital for Wimbledon, Merton, and district, which is open to local architects as well as a limited number of others with special hospital building experience. The cost is not to exceed £6,000. The hospital is to contain three wards, one for men and one for women, each of these having eight beds, while the third ward will be for children and will have four beds. In addition there are to be four single-bedded rooms, one of which may be used for a paying patient. There will be an operating theatre, with an ante-room which may be used for anæsthetising patients and for sterilising purposes, a small dispensary, and a mortuary. Accommodation will be provided for a nursing staff consisting of one matron, two staff nurses, and two probationers, and the usual domestic staff, the kitchen, scullery, larder, pantry, and coal store being situated on the ground floor. The building throughout will be lighted by electricity, but a gas jet will also be provided in each room. A condition of the competition is that provision must be made for extending the accommodation at a future date to forty beds, and for the possible addition of a laundry, this extension to interfere as little as possible with the original building.

### NORTH STAFFORDSHIRE INFIRMARY.

Mr. Henry Roscoe, the senior assistant medical officer and deputy superintendent at Cheddleton Asylum, who has been appointed to the position of secretary and house governor to the North Staffordshire Infirmary, is an M.R.C.S., L.R.C.P., and D.P.H., and qualified in 1894. He has held the appointments of house surgeon at Ancoats Hospital in 1895, assistant resident medical officer at Manchester Union Infirmary the following year, and resident medical officer at the Dyffryn Aled branch of the Cheadle Royal Asylum in 1897-8. In the latter year he proceeded to South Africa, taking up an appointment as surgeon-captain with the British South Africa Mounted Police, and afterwards holding the position of district surgeon at Gwanda, Rhodesia, until 1903. On his return to this country he was for two years engaged in research work at the Public Health Laboratories, Manchester, and acted as junior assistant to Professor Delepine. He was appointed junior assistant medical officer at Cheddleton Asylum in April 1907, and in November of the same year became senior assistant medical officer and deputy superintendent. Mr. Roscoe has had a large experience of hospital administration. The Gwanda District Hospital was built in accordance with his ideas and requirements, and thereafter, for five years, he had sole charge of the general management and expenditure. At the request of the late Mr. Cecil Rhodes he surveyed a site for Gwanda township, and Mr. Rhodes accepted, and carried out, all his suggestions. During the Boer War, at the request of Sir Arthur Lawley, Administrator of Matabeleland, he assisted Colonel Plumer's medical staff during a three months' period of stress, and received special thanks for his services. While at Cheddleton Asylum Mr. Roscoe has extended his experience of hospital administration, every department at this large institution being under the direct supervision of the medical superintendent, to whom Mr. Roscoe has acted as deputy, and during the latter's absence, on vacation leave, he has had sole control of the institution.

### THE QUEEN AND A GLASGOW HOSPITAL.

Last week a meeting was held at the Glasgow Maternity and Women's Hospital in Rottenrow to discuss the proposal to hold a fancy-dress ball in aid of the institution. Mrs. M'Innes Shaw, who presided, reported that Her Majesty the Queen, who is the patron of the hospital, had been asked, through the Lord Provost, if she would confer the further honour on the hospital of accepting an album containing photographs of the children in costume who took part in the forthcoming ball as a souvenir of the occasion. The following reply from Buckingham Palace, dated October 21, had been received :—

My Lord Provost.—I have had the honour of submitting your letter to the Queen. I am desired by Her Majesty to say that it will give her great pleasure to accept the album containing photographs of the children in costume in connection with the ball to be held in aid of the new Glasgow Maternity and Women's Hospital, and I am to ask you to kindly convey Her Majesty's thanks and good wishes for the success of the charity.—I am, my Lord Provost, your obedient servant, Sidney Grenville.

A representative committee was afterwards formed, with Mrs. M'Innes Shaw as convener, and the date of the ball was fixed for Friday, January 14, in St. Andrew's Halls.

### IN AID OF BRISTOL CHARITIES.

The charity trip run by Messrs. P. and A. Campbell, Ltd., by their steamer *Westward Ho* (on September 27) for the benefit of the local charities realised £32 2s. 6d. This amount was divided as follows : Bristol General Hospital, £7 7s. ; Bristol Royal Infirmary, £7 7s. ; Bristol Eye Hospital, £3 13s. 6d. ; Bristol Royal Hospital for Sick Children and Women, £3 13s. 6d. ; Clifton Dispensary, £10 1s. 6d. This being the twenty-first annual charity trip, it may be interesting to know that since these trips were started by Messrs. Campbell the total handed over to the charities amounts to £611 11s. 1d.

### THE ABERDEEN CITY HOSPITAL.

At the last meeting of the Public Health Committee of the Aberdeen Town Council it was agreed to accept the lowest offer for the electric lighting of the hospital—that of £610 by Messrs. Blaikie and Sons, Aberdeen. It was reported that, in reference to this matter, the Contractors' Association had abandoned their threatened interdict. A rearrangement of the hours for visitors at the hospital was recommended, the hours now proposed being from 5.15 to 6 o'clock on Tuesday afternoons, and from 4 to 4.45 on Saturday afternoons.

